# Extrahepatic metastases as initial manifestations of hepatocellular carcinoma: an Egyptian experience

**DOI:** 10.1186/s13000-015-0313-1

**Published:** 2015-06-30

**Authors:** Thanaa El. A. Helal, Nehal A. Radwan, Mohamed Shaker

**Affiliations:** Departments of Pathology, Faculty of Medicine, Ain Shams University, Ramses Street- New Faculty Bldg. -5th floor, P.O. # 11566 Cairo, Egypt; Departments of Radiology, Faculty of Medicine, Ain Shams University, Ramses Street- Radiology department, Ain Shams University Hospital, P.O. # 11566 Cairo, Egypt

**Keywords:** Hepatocellular carcinoma, Metastases, Initial presentation, Immunohistochemistry

## Abstract

**Background:**

The incidence of hepatocellular carcinoma (HCC) in Egypt has markedly increased in the recent years, mainly due to the high incidence of hepatitis C virus (HCV) infection. Consequently, the frequency of metastatic HCC has also increased. The current study presents a series of 47 patients who were initially diagnosed as metastatic HCC.

**Methods:**

Forty seven patients with the diagnosis of extrahepatic metastases of HCC at initial presentation were included in the study. The sites of metastases were bones (17), lymph nodes (9), soft tissue (7), omentum (7), maxillary sinus (2), adrenal gland (2), brain (2) and skin (1). The diagnosis of metastatic HCC was confirmed by immunohistochemistry.

**Results:**

The patients included in the study were 38 males and 9 females, ranging from 40 to 80 years (median 60 years). All patients were HCV-positive and 36 were cirrhotic. The diagnosis of primary HCC was confirmed in all cases, based on the typical hypervascular radiological features and/or high serum α-fetoprotein concentration, or histologic examination of liver biopsy.

**Conclusion:**

Metastasis of HCC should be put into consideration when evaluating metastatic carcinoma with unknown primary. This is of particular importance in the Egyptian population who has the highest prevalence of HCV infection in the world.

## Background

The incidence of hepatocellular carcinoma (HCC) shows a significant geographic variation worldwide. In Southeast Asia and Sub-Saharan Africa, the incidence is more than 15/100.000 population per year due to the high prevalence of chronic hepatitis B virus (HBV) infection and chronic hepatitis C virus (HCV) infection [1]. Although HCC has been relatively uncommon in the Western countries, recent evidence shows an increasing rate in these areas [2–4]. In Egypt, hospital-based studies have reported that the annual proportion of HCC showed a significant rising trend from 4 % in 1993 to 7 % in 2002 of chronic liver disease patients [5]. This high incidence of HCC in Egypt is explained by the increasing prevalence of HCV infection [6]. Unfortunately, Egypt has the highest prevalence of HCV infection in the world with 13.8 % of the population infected [7].

Extrahepatic metastatic spread of HCC occurs in about 50 % of cases, most often to the lungs, lymph nodes and bones [8]. The clinical presentations of patients with HCC were mostly concerned with the manifestations of the primary tumor and the metastatic presentation is a later event [9]. In the recent years, the metastatic behavior of HCC has shown two interesting new features. First, is the spread to unusual sites as soft tissue [10, 11], maxillary sinus [12] and orbit [13]. Second, is the identification of the metastatic lesion before the primary one is diagnosed [14–18].

Budhu *et al.* [19] have reported that many of the metastasis-promoting genes are embedded in the primary tumors and that the ability to metastasize may be an inherent quality of the tumor from the beginning. The condition of the liver parenchyma, the degree of viral hepatitis mediated liver damage and the genetic makeup of individuals may have a significant role to play in the development of metastases. Accordingly, it is possible that the geographic variation in the gene expression profile of liver parenchyma among HCC patients and/or the differences in the genetic makeup among populations may lead to epidemiologic variations in the metastatic behavior of HCC. This concept stimulates us to investigate a cohort of Egyptian patients who were initially presented with extrahepatic metastases.

The value of this work is bifold: first, it includes a relatively large number of cases who initially presented with metastatic HCC before the diagnosis of primary HCC was established. Similar studies are available in the literature only as case reports or case series except for four studies [14, 20–22]. Second, this study included Egyptian patients; all of whom were HCV-positive. To our knowledge, no similar reports are available in the literature.

## Methods

**Fig. 1:**
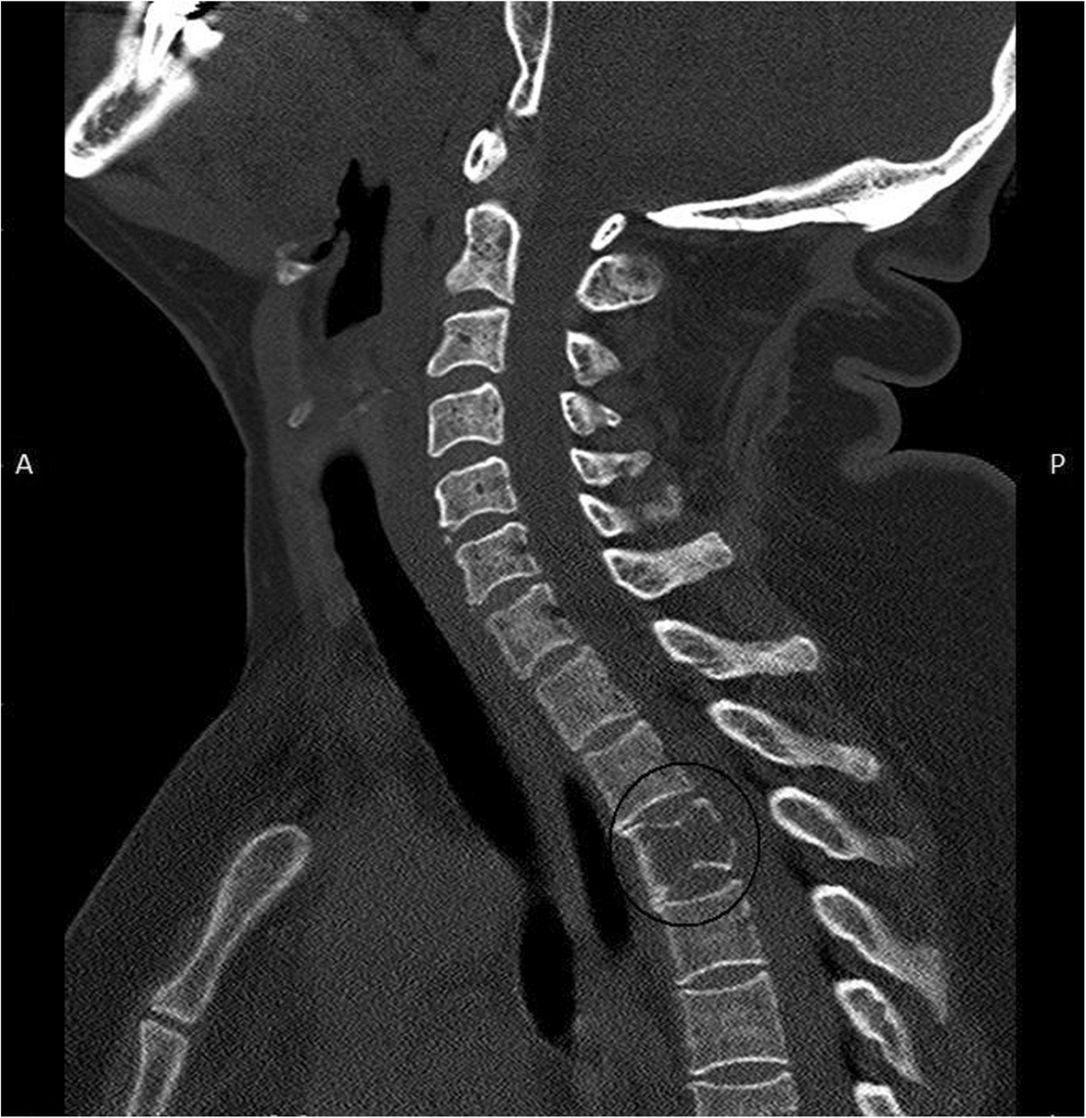
Computed tomography (sagittal reconstruction) showing metastatic mass from HCC infiltrating body of third dorsal vertebra (circle)
- *Lymph node metastases (9 cases)*
  Six cases were located in the abdominal lymph nodes and 3 were situated in the retroperitoneal, inguinal and supraclavicular lymph nodes. Guided needle or excisional biopsies from these sites were performed.
- ***Soft tissue metastases (7 cases)***
  These metastases were located in the anterior chest wall (4 cases), shoulder (1 case), back, deep to angle of scapula (1 case) and forearm (1 case). CT scan revealed a large soft tissue mass with no underlying bone destruction. Needle biopsies were taken for pathological assessment.
- ***Omental metastases (7 cases)***
  The main complaint of these cases was abdominal pain, CT scan showed hypoechoic solitary mass in 2 patients and multiple nodules in 5 patients.
- ***Adrenal gland metastases (2 cases)***
  The patients presented with lumbar pain. Abdominal ultrasound detected a retroperitoneal mass close to the kidney. CT scan with contrast was done and revealed an enhancing adrenal mass from which needle biopsy was taken.
- ***Brain metastases (2 cases)***
  Both patients complained of headache and vomiting, CT scan showed a space occupying lesion in the left parietal lobe and right fronto-temporal area with homogeneous enhancement. Craniotomy was done and the tumor was excised.
- *Maxillary sinus metastases (2 cases)*
  One of the two patients presented with severe bleeding after tooth extraction. The other had felt pain on occlusion and movement of the right upper molar, CT examination of both cases revealed soft tissue density infiltrating the adjacent bones and muscles.
- ***Skin metastases (1 case)***
  One patient presented with a cutaneous non ulcerating nodule on his hand. Radiography and CT scan of the hand suggested a subcutaneous metastasis.

A total of 47 cases, pathologically confirmed as metastatic HCC at initial presentation with no known liver primaries were retrieved retrospectively from the files of the pathology departments at Ain Shams University hospitals during the period 2001–2010. The medical records of these patients were examined to collect all relevant clinical, radiological and laboratory data. According to the metastatic site; these cases were categorized as follows:

- ***Bone metastases (17 cases)***
  The most common site of bone metastases was the ribs (6 cases) followed by the mandible (4 cases). The remaining cases were located in the humerus, femur, iliac bone and vertebral bodies (Fig. 1) (one or two cases for each site). They were clinically and radiologically suspected as primary or secondary malignant neoplasm. Biopsies were taken from all lesions.

### Histologic and immunohistochemical examination

Tissue biopsies from all 47 patients were examined by the conventional Hematoxylin and Eosin (H&E) staining method. Paraffin embedded tissue sections were also processed for immunohistochemistry using standard labeled streptavidin- biotin-peroxidase complex technique. Briefly, tissue sections were deparaffinized and hydrated in xylene and descending grades of alcohol. Antigen retrieval was performed by treating the tissue sections with citrate buffer, pH 6.0 for 10 min in a 700-W microwave oven. The endogenous peroxidase activity was blocked by incubating the slides in 3 % hydrogen peroxide for 5–10 min. This is followed by incubation with the primary antibody (hepatocyte paraffin antigen-1 (HepPar-1), α fetoprotein (AFP), CD34, CK7 and CK20 (Table 1)). The antibody reaction was detected with the avidin-biotin detection kit using diaminobenzidine (DAB) as chromogen. Proper positive control was applied, while negative control was done using the same tissues, omitting the primary antibody. HepPar −1 is known as one of the most specific and sensitive markers for HCC [23]. AFP is also a specific marker of HCC [24].

**Table 1:** Primary antibodies used for immunohistochemistry in the current study

Antibody	Code no.	Dilution	Type	Company	Country
HepPar-1	MS-18100-R7	Ready to use	Monoclonal Mouse	Lab Vision	CA, USA
AFP	A 0008	1:400–1:800	Polyclonal rabbit	Dako	Denmark
CD34	M7165	1:50	Monoclonal Mouse	Dako	Denmark
CK7	M7018	1:100	Monoclonal Mouse	Dako	Denmark
CK20	M7019	1:50	Monoclonal Mouse	Dako	Denmark

*HepPar-1* hepatocyte paraffin antigen-1, *AFP* α fetoprotein

### Retrograde data collection

When the diagnosis of metastatic HCC was confirmed by immunohistochemistry in all 47 patients included in the study, the data base of those patients were reviewed to investigate for the presence of primary HCC. The following data were collected:

- Clinical data: Patients age, gender, history of liver disease, presence of hepatomegaly or other manifestations of HCC.
- Radiologic data: Abdominal ultrasonography and/or CT were performed.
- Laboratory data: Serum AFP level as measured by radioimmunoassay (cut off level 20 ng/ml), hepatitis B surface antigen (HBsAg), hepatitis C antibody and serum virus C RNA which was detected by real time PCR.
  Finally, the diagnosis of primary HCC was based on the presence of hepatic focal lesion(s) with typical hypervascular radiological features and/or high serum AFP level or pathologic examination of liver biopsy [21]. The study was carried out with full local ethics approval.

## Results

The current study included 38 males and 9 females with M: F ratio 4.2:1. Their ages ranged from 40 to 80 years (median 60 years). The characteristics of the 47 patients are given in Table 2.

**Fig. 2:**
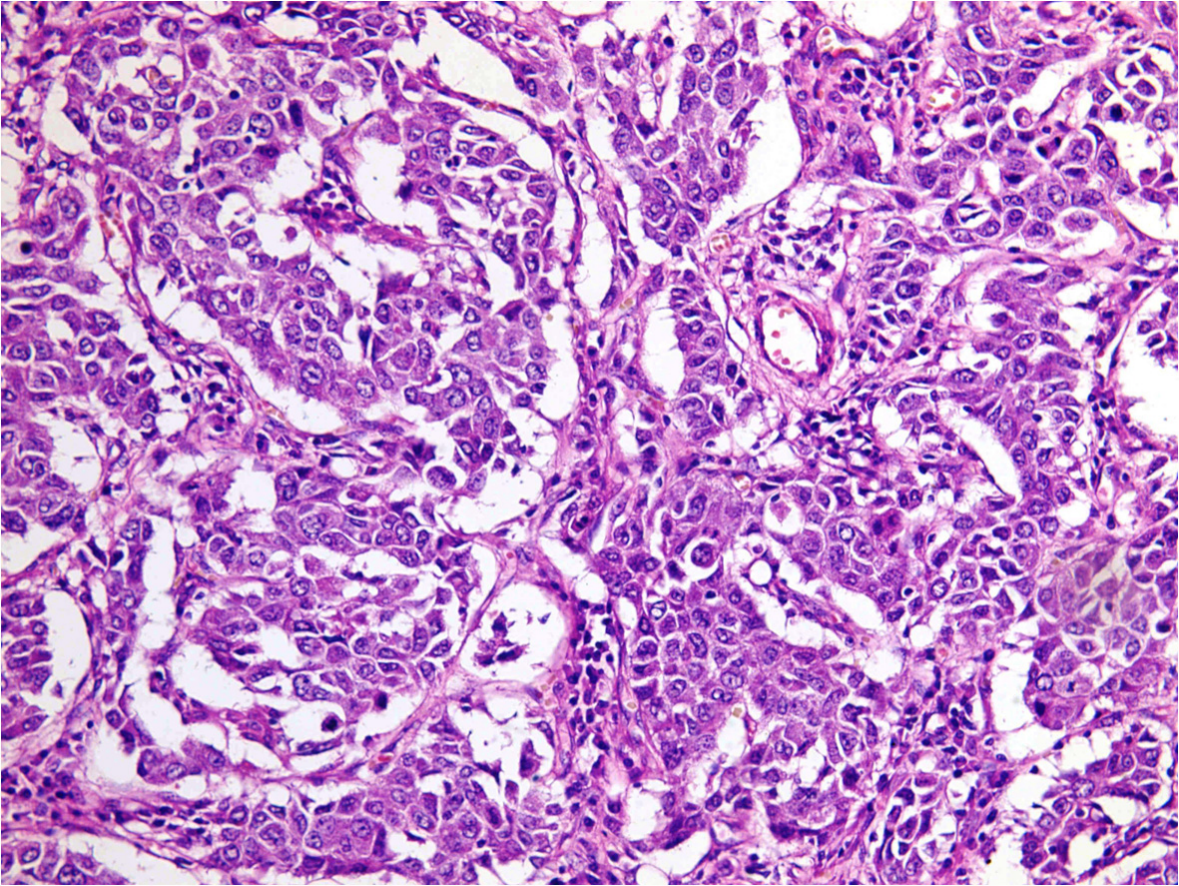
A case of metastatic hepatocellular carcinoma. Note the trabecular pattern of growth with pleomorphic, hyperchromatic nuclei; (H&E, original magnification × 400)

**Fig. 3:**
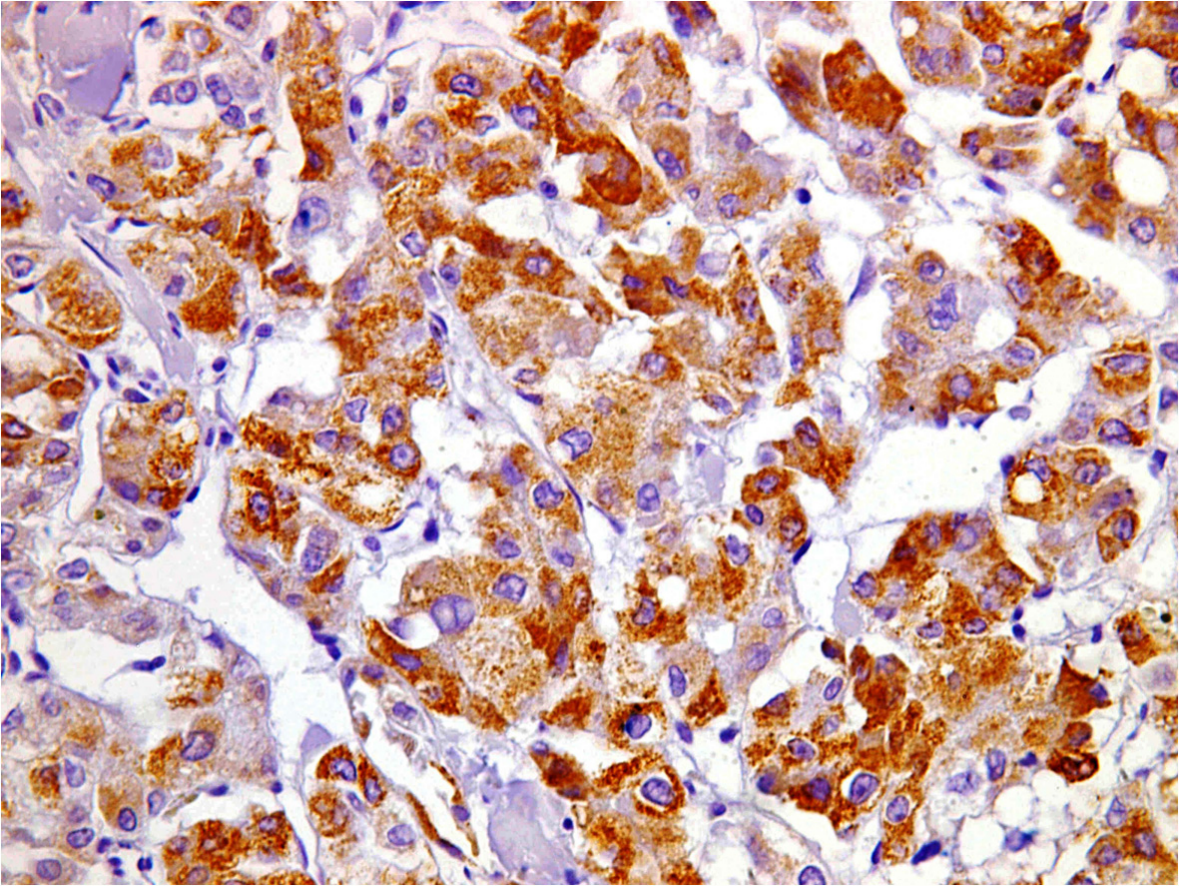
A case of metastatic hepatocellular carcinoma with strong and diffuse cytoplasmic positivity for HepPar-1;(immuoperoxidase, original magnification × 400)

**Fig. 4:**
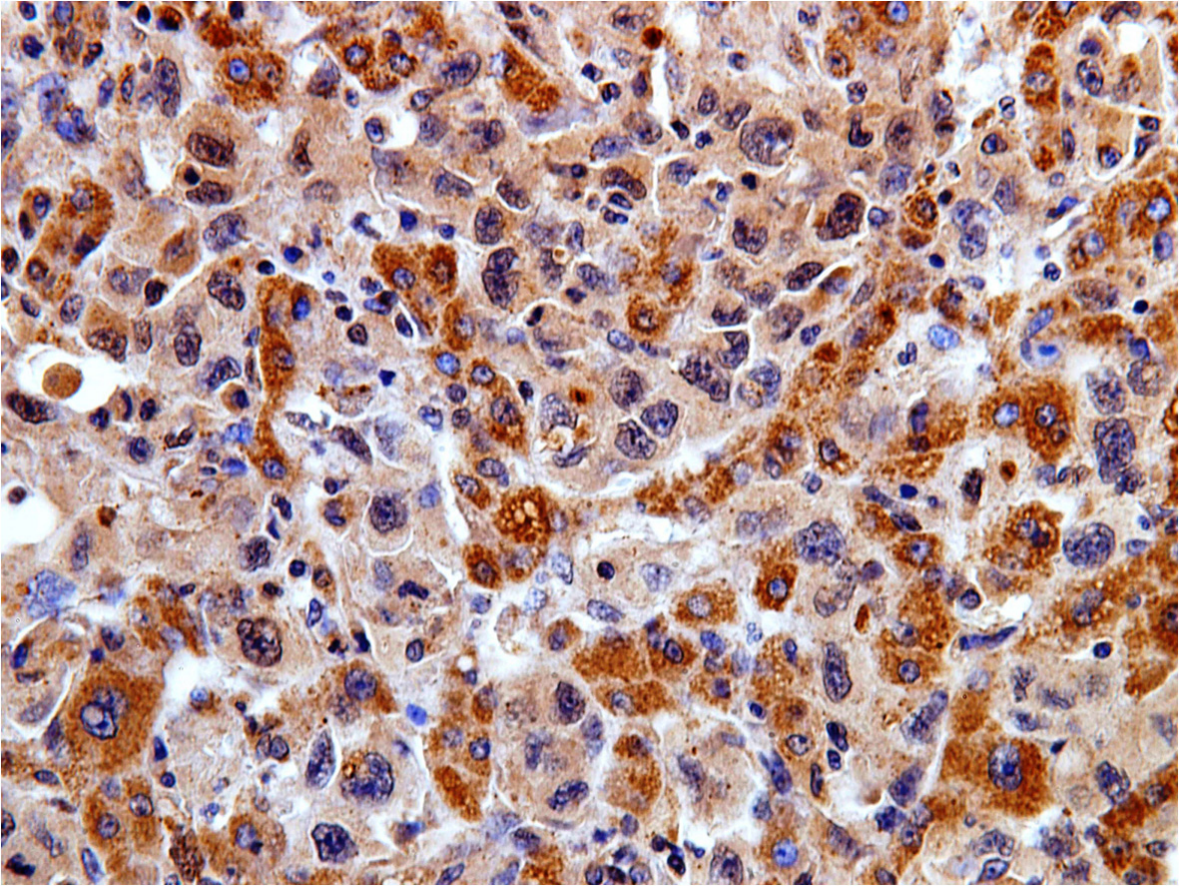
A case of metastatic hepatocellular carcinoma with positive cytoplasmic immunostaining for AFP;(immuoperoxidase, original magnification × 400)

**Fig. 5:**
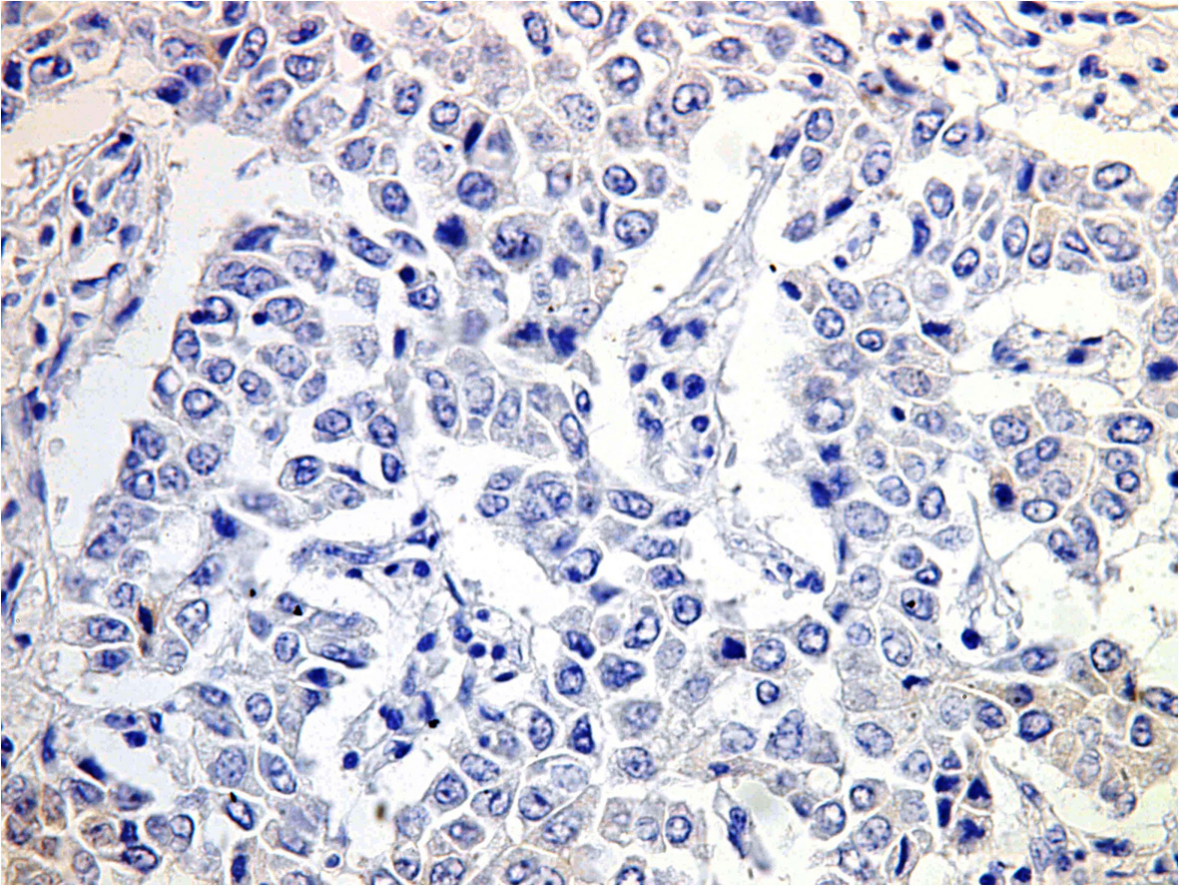
A case of metastatic hepatocellular carcinoma with negative immunostaining for CK 20;(immuoperoxidase, original magnification × 400)

- ***Clinical findings***: All 47 patients had no clinical features suggestive of HCC. Their initial presentation was in the form of extrahepatic mass lesion as described in the material and methods.
- ***Radiologic examination of the liver***: Thirty six patients (76.6 %) had cirrhosis. Hepatic focal lesions were detected in all patients. These lesions were solitary in 38 patients (80.9 %) ranging from 8 mm to 2.5 cm in diameter. The remaining 9 patients, (19.1 %) had multiple focal lesions varying between 5 mm and 1.5 cm.
- ***Laboratory findings***: All patients were positive for HCV by real time PCR and one patient was also positive for hepatitis B virus antigen. Twenty two of the 47 patients (46.8 %) showed elevated serum AFP level.
- ***Histology***: All metastatic lesions showed malignant tumor tissue which consists of large hepatocyte-like cells with moderate nuclear atypia and prominent mitotic activity. The cells are mostly arranged in trabecular or sinusoidal pattern, which is very reminiscent to that seen in primary HCC (Fig. 2). So, the histologic diagnosis was metastatic carcinoma of possible hepatocellular origin.
- ***Immunohistochemistry*:** In all cases, the tumor cells showed granular cytoplasmic positivity for HepPar-1 (Fig. 3) and/or AFP (Fig. 4). For HepPar-1, the staining was diffuse and strong in 28(59.6 %) cases while AFP revealed focal and weak staining in 15 (32 %) cases. CD34 highlighted the rich vasculature of the tumor tissue and staining of the sinusoidal endoethelial cells. Staining for CK7 and CK20 yielded negative results (Fig. 5).

**Table 2:** Characteristics of 47 hepatocellular carcinoma patients with initial extrahepatic metastases

	Number	Percent
Male/Female	38/9	
Age range	40–80 years	
Median age	60 years	
HCV	47	100
HBV	1	2.1
Elevated AFP	22	46.8
Cirrhosis	36	76.6
Hepatic focal lesion:		
-Solitary	38	80.9
-Multiple	9	19.1
Site of metastases:		
- Bones	17	36.2
-Lymph nodes	9	19.1
- Soft tissue	7	15
- Omentum	7	15
-Maxillary sinus	2	4.2
-Adrenal gland	2	4.2
-Brain	2	4.2
-Skin	1	2.1
Pattern of HCC growth :		
-Trabecular (plate like)	8	17 %
- Pseudoglandular (acinar)	2	4.3 %
-Solid	10	21.3 %
-Mixed patterns	27	57.4 %
Grade:		
- Well differentiated	0	0 %
- Moderately differentiated	17	36.2 %
- Poorly differentiated	30	63.8 %

*HCV* hepatitis C virus, *HBV* hepatitis B virus, *AFP* α fetoprotein

## Discussion

Population-based cancer registry in Egypt reported that the incidence of HCC in Gharbiah (one of the Egyptian provinces) in the period from 1999 to 2003 was 3.5 times higher than that reported in the United States [25]. These data indicate that HCC represents a major health problem in Egypt and emphasize the importance of enhancing the early detection programs of primary HCC. In this context, we should be aware that about 5 % of HCC may be presented as extrahepatic metastases for the first time [21, 24].

The primary purpose of the present work was to include a relatively large number of cases who initially presented with metastatic HCC before the diagnosis of primary HCC was established. By reviewing the literature, we found that nearly all publications on HCC diagnosed initially as extrahepatic metastases are case reports or case series except for four studies [14, 20–22] (Table 3). Most importantly, the current study included Egyptian patients; all of whom were HCV-positive. To our knowledge, no similar reports are available in the literature.

**Table 3:** Summary of reported cases of hepatocellular carcinoma initially presented with extrahepatic metastases

Authors name	Year of publication	No of patients	Age range (Median)	Male/Female	Pattern of growth of HCC	Metastatic anatomic region
Liaw *et al.* [20]	1989	20	26–64 (50)	16/4	Predominantly trabecular pattern	Spines, ribs, skull, scapula, pelvis, long bone, sternum, clavicle.
Uka *et al. *[22]	2007	151	21–82 (64)	117/34	Not mentioned	Lung, lymph nodes, bones, adrenal gland, peritoneum, pancreas, nasal passages.
Yoo *et al.* [21]	2011	251	18–85 (51)	212/39	Not mentioned	Lung, lymph node, bones, adrenal gland, others.
The present study	2015	47	40–80 (60)	38/9	Predominantly mixed pattern	Bones, lymph nodes , soft tissue, omentum, maxillary sinus, adrenal gland, brain, skin.

The largest series included 20 Chinese patients presented with bone metastases [20]. Another large study of 251 patients with extrahepatic metastases, at initial diagnosis of HCC was performed in Korea [21]. However, this study was concerned with the therapeutic modalities and clinical outcome of the studied patients. No immunohistochemistry was performed. The current study applied 5 antibodies for the immunohistochemical confirmation of the diagnosis. Moreover; all HCC patients included in our work were HCV-related as compared to the Korean study which included a heterogeneous group of HCC patients, related to HBV, HCV, alcohol or others. Variations in the etiology of HCC may have an impact on its metastatic behavior.

The present study included 47 cases with HCC who were initially presented by extrahepatic metastases. This number constitutes about 6.5 % of the total number of HCC cases encountered during the period 2001–2010 (Total number of HCC cases = 720). This percentage approximated that of Yoo *et al.* [21] who obtained a ratio of 5.4 %.

Furthermore; in our study, the male to female ratio was 4.1:1 and the median age was 60 years. This gender and age distribution is similar to that reported for primary HCC in Egypt [25]. On the other hand, the gender distribution in our study approximates that found in the aforementioned Asian reports [20, 21]. Nevertheless, the median age in both studies was 50 years which is 10 years earlier than that found in the current study. The older age of our patients may be attributed to the fact that all of them were HCV-associated. HCV-related HCC usually occurs after the development of cirrhosis. The average time from infection to onset of cirrhosis is 13–25 years and time to onset of liver cancer is 17–31 years [26].

Most of the studies evaluating the sites of metastases of HCC did not differentiate between those occurring in patients already diagnosed as having primary HCC and other cases in whom metastases were the initial presentation with silent primary HCC [22, 27–29]. Fukutomi *et al.* [30] reported that the incidence of bone metastases from HCC is estimated to be 2–16 % depending on the prevalence of the primary disease in the population.

In the current series, the most common site of metastasis was the skeleton (36.2 %), followed by lymph nodes (19.1 %). This contrasts with the results of Yoo *et al.* [21] who found that lymph node and bone metastases were detected in 37.5 and 18 % of their cases respectively. This difference may be due to variation in patient selection. Alternatively, this contrast between our findings and those of Yoo *et al.* may reflect differences in the genetic profile and biologic behavior of Egyptian and Asian patients with HCC. In fact, the higher frequency of bone metastases as compared to lymph node metastasis in the present study was expected because of the high vascularity of HCC and the liver itself [31]. Last but not least, it is important to refer to the report of Olubuyide [32] who claimed that the pattern of metastases of HCC from the cirrhotic liver is different from that of non-cirrhotic liver i.e. HCC from cirrhotic liver has few skeletal metastases. Since about 70 % of our cases were cirrhotic, this speculation of Olubuyide seems not compatible with our results.

By collecting the metastases to the oro-maxillofacial region in the current study, they were totally 6 cases (4 mandible and 2 maxillary sinus). This finding supports the previous reports that documented the high frequency of HCC metastatic to the head and neck region as an initial presentation of the disease [33, 34].

Metastatic HCC to the oro-maxillofacial region is generally believed to occur via the lung [35]. However, all our 6 cases with oro-maxillofacial metastases did not have lung metastases by diagnostic imaging. It has been suggested that metastases could occur without lung metastases either by vertebral and azygos vein system or by lymphatic system. Spread via the former route is the preferable one in case of liver cirrhosis [36]. Since all 6 cases with oromaxillofacial metastases had liver cirrhosis, metastasis by vertebral vein system seems more likely.

## Conclusions

In conclusion, the current study underscores that pathologists and surgeons, especially orthopedic and head and neck surgeons, must be prepared to consider the possibility of metastatic HCC higher on the list of their differential diagnosis. We also emphasize the importance of adding HepPar-1 and AFP to the panel of immunohistochemical markers applied for identification of the primary site of metastatic carcinoma. This is of particular importance in our country which has a very high incidence of HCC.

## Abbreviations

HCC: Hepatocellular carcinoma
HBV: Hepatitis B virus
HCV: Hepatitis C virus
CT: Computed tomography
H&E: Hematoxylin and Eosin
HepPar-1: Hepatocyte paraffin antigen-1
AFP: α fetoprotein
HBsAg: Hepatitis B surface antigen
